# Supplementary material for: Small molecule targeting r(UGGAA)n disrupts RNA foci and alleviates disease phenotype in Drosophila model
Source: Nat Commun. 2021 Jan 11;12:236. doi: 10.1038/s41467-020-20487-4 (PMC7801683; doi:10.1038/s41467-020-20487-4)
Supplement: Supplementary file 1 — Supplementary Information [file 41467_2020_20487_MOESM1_ESM.pdf]

Supplementary Information

Small molecule targeting r(UGGAA)$_n$ disrupts RNA foci and alleviates disease phenotype in *Drosophila* model

Tomonori Shibata,[1] Konami Nagano,[2] Morio Ueyama,[3] Kensuke Ninomiya,[4] Tetsuro Hirose,[4,5] Yoshitaka Nagai,[3] Kinya Ishikawa,[6] Gota Kawai,[2] and Kazuhiko Nakatani[1]*

[1] Department of Regulatory Bioorganic Chemistry, The Institute of Scientific and Industrial Research (ISIR), Osaka University, Ibaraki, Japan

[2] Department of Life and Environmental Sciences, Faculty of Engineering, Chiba Institute of Technology, Chiba, Japan

[3] Department of Neurotherapeutics, Osaka University Graduate School of Medicine, Suita, Japan

[4] Graduate School of Frontier Biosciences, Osaka University, Suita, Japan

[5] Institute for Genetic Medicine, Hokkaido University, Sapporo, Japan

[6] Center for Personalized Medicine for Healthy Aging, Tokyo Medical and Dental University, Tokyo, Japan

* Correspondence to Kazuhiko Nakatani (nakatani@sanken.osaka-u.ac.jp)

Contents

**Supplementary Figure 1**	Chemical structures of 20 compounds (LC-1–20) in in-house chemical library.	P 3
**Supplementary Figure 2**	SPR analysis of the binding of LC-1–20 to r(UGGAA)$_9$-, r(UAGAA)$_9$- and r(UAAAA)$_9$-immobilized surfaces.	P 4
**Supplementary Figure 3**	EMSA to confirm interactions of r(UGGAA)$_9$ with molecules.	P 5
**Supplementary Figure 4**	EMSA to confirm interactions of r(UGGAA)$_9$ with molecules.	P 6
**Supplementary Figure 5**	Thermal melting curves of r(UAGAA)$_9$ and r(UAAAA)$_9$ in the absence and presence of NCD.	P 7
**Supplementary Figure 6**	Thermal melting curves of RRA/RRA internal loop-containing RNA duplexes in the absence and presence of NCD or QCD.	P 8
**Supplementary Figure 7**	Thermal melting curves and CD spectra of UGGAA-UGGAA pentad-containing hairpin RNA in the presence of NCD.	P 9
**Supplementary Figure 8**	ESI-TOF-MS analysis of UGGAA-UGGAA pentad-containing hairpin RNA with NCD.	P 10
**Supplementary Figure 9**	Examples of intermolecular NOEs of SCA31RNA and NCD.	P 11
**Supplementary Figure 10**	EMSA to confirm the interaction of r(UGGAA)$_9$ with RNA FISH probe in the absence and presence of NCD and WST-1 assay and qPCR of r(UGGAA)$_{76}$ in the absence and presence of ligands.	P 12
**Supplementary Figure 11**	RNA FISH and IF images of HeLa cells expressing r(UGGAA)$_{76}$ in the absence and the presence of NCD or QCD stained by r(UGGAA)$_{76}$-FISH and IF using anti-TDP-43 antibodies.	P 13
**Supplementary Figure 12**	RNA FISH and IF images of HeLa cells after thermal stress exposure in the absence and the presence of NCD or QCD stained by HSATIII-FISH and IF using anti-SRSF9 antibodies.	P 14
**Supplementary Figure 13**	ITC measurements for the binding of NCD to CGG/CGG-containing hairpin DNA.	P 15
**Supplementary Table 1**	NMR constraints and structure statistics.	P 16
**Supplementary Table 2**	All-atom structure validation by MolProbity	P 17–18
**Supplementary Table 3**	Primer sequences used in quantitative and semi-quantitative RT-PCR	P 19

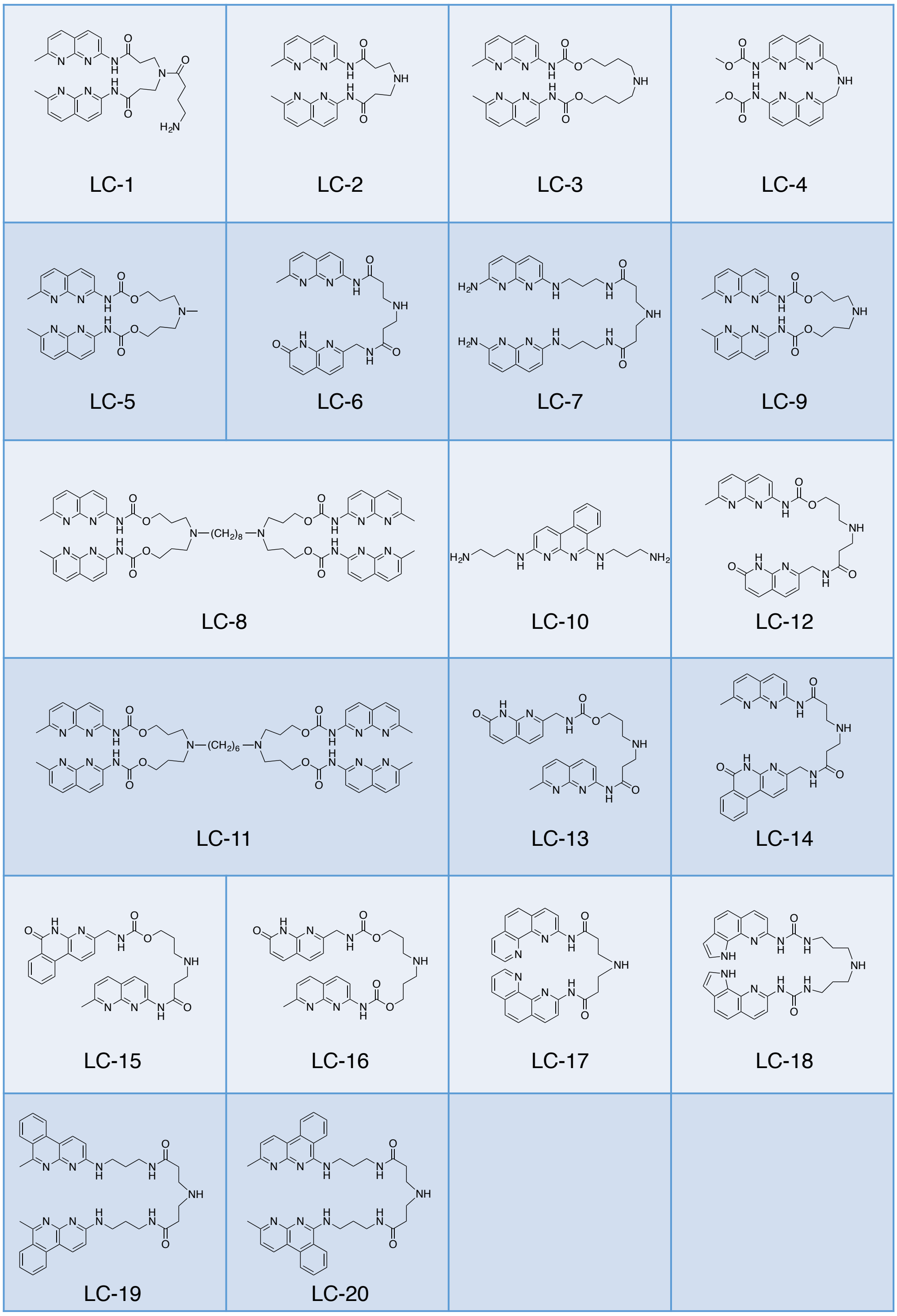

Supplementary Figure 1

Chemical structures of 20 compounds (LC-1–20) in in-house chemical library.

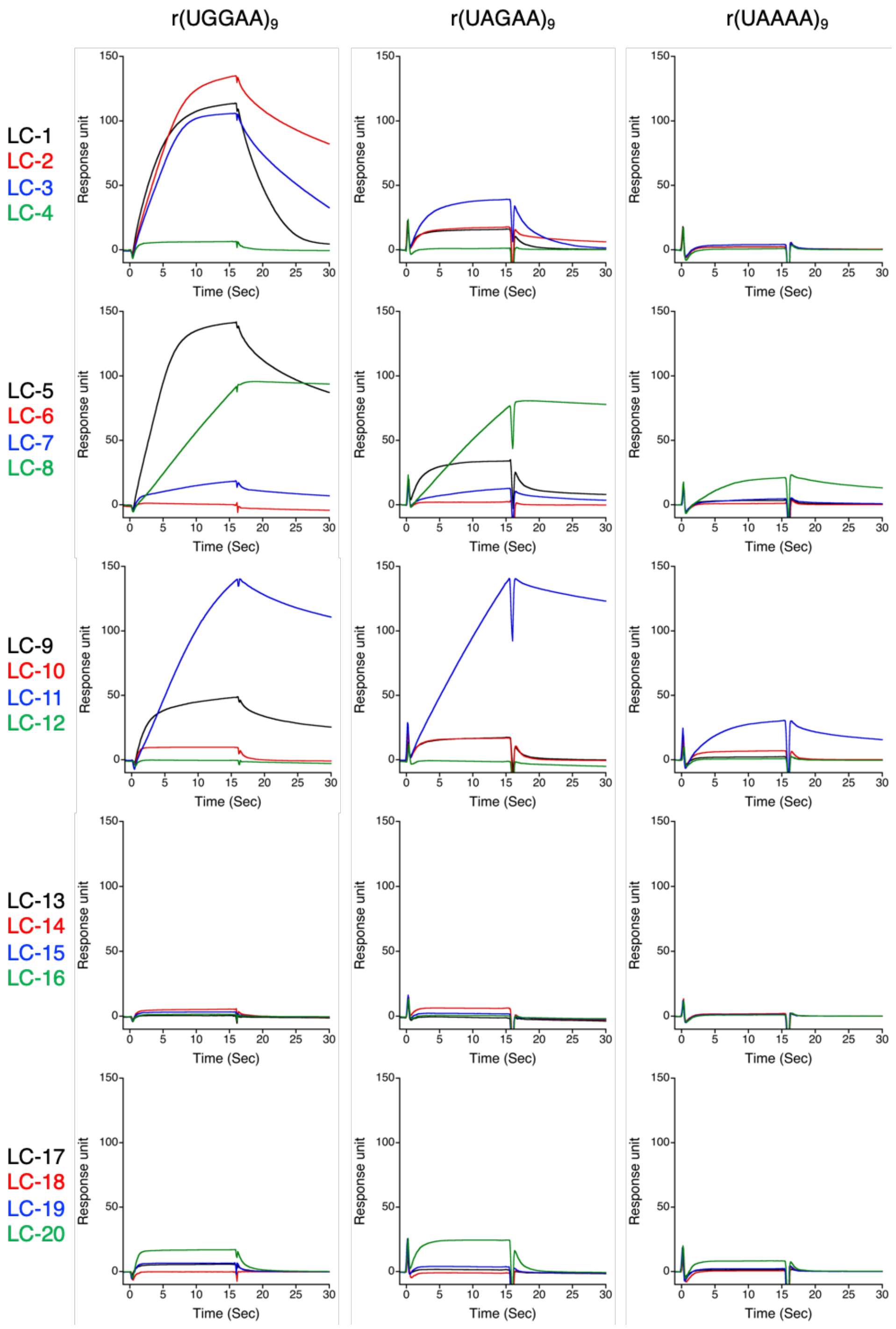

Supplementary Figure 2

SPR analysis of the binding of LC-1–20 to r(UGGAA)$_9$-, r(UAGAA)$_9$- and r(UAAAA)$_9$-immobilized surfaces. Compound concentration was 500 nM. The amount of 5’-Biotin-labelled r(UAGAA)$_9$, r(UAGAA)$_9$ and r(UAAAA)$_9$ immobilized on the SA sensor chip was 586, 658, and 529 RU, respectively.

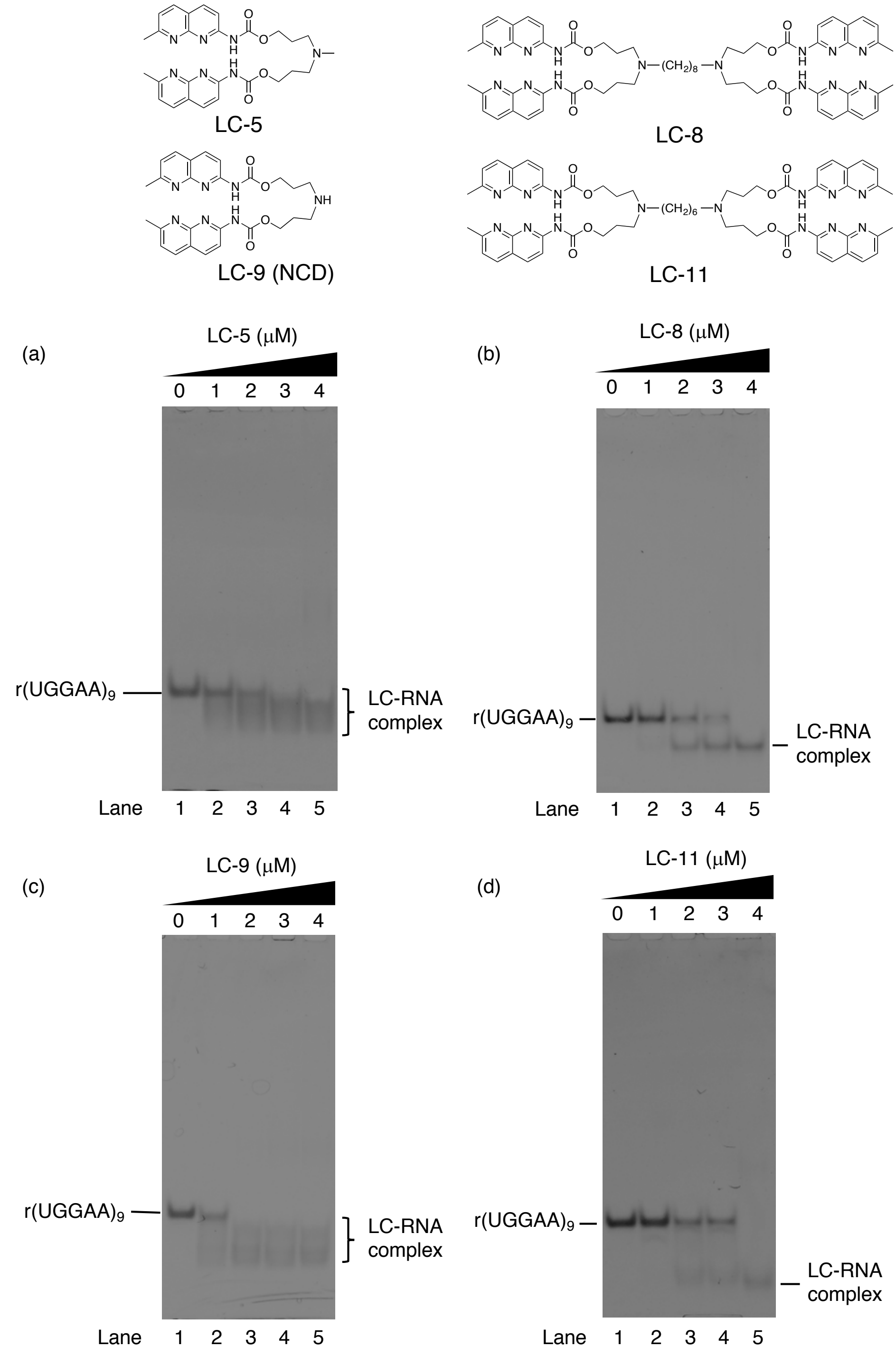

Supplementary Figure 3

EMSA to confirm interactions of r(UGGAA)$_9$ with (a) LC-5, (b) LC-8, (c) LC-9, and (d) LC-11. RNA concentration: 200 nM. Compound concentration was 0, 1, 2, 3 and 4 μM.

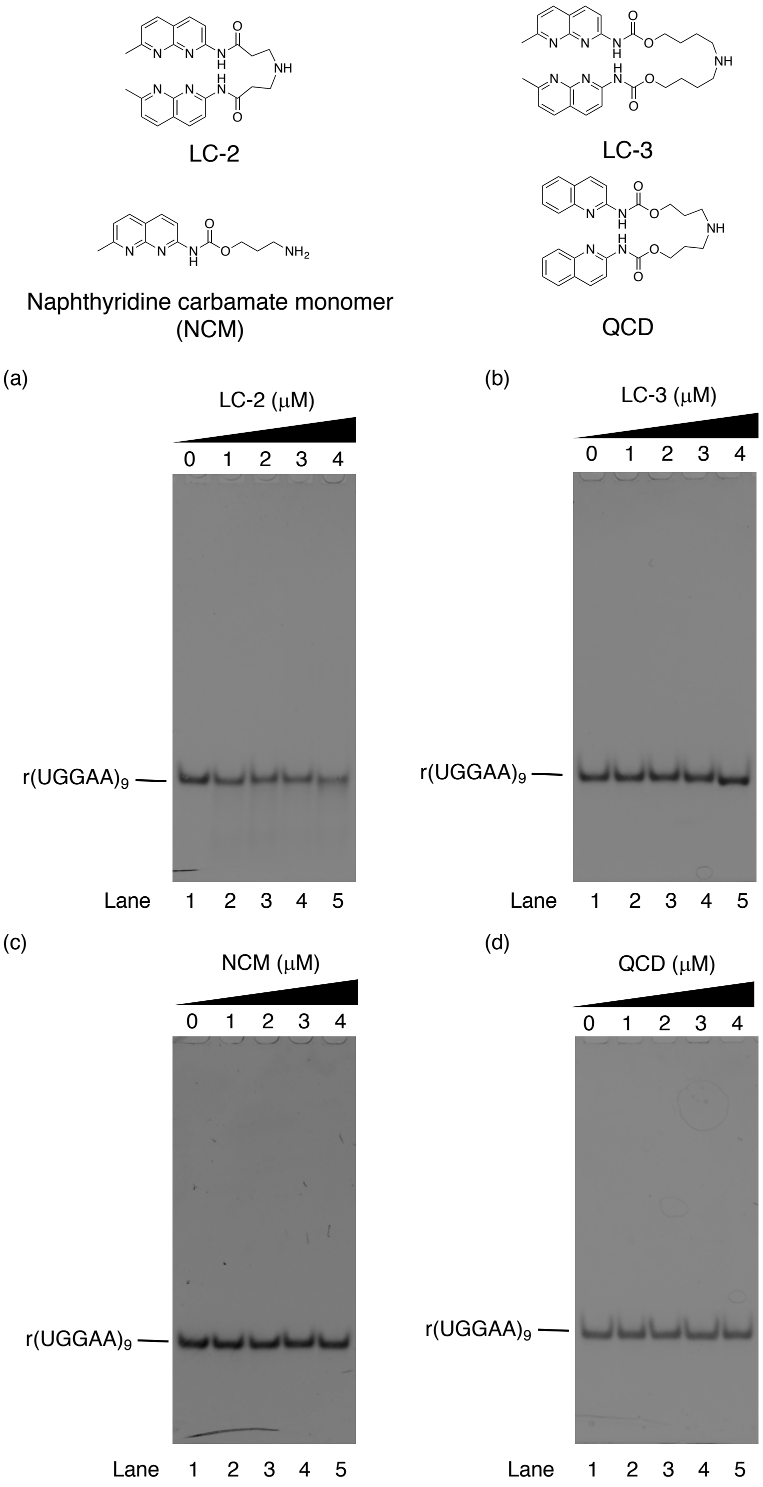

Supplementary Figure 4

EMSA to confirm interactions of r(UGGAA)$_9$ with (a) LC-2, (b) LC-3, (c) NCM, and (d) QCD. RNA concentration: 200 nM. Compound concentration was 0, 1, 2, 3 and 4 μM.

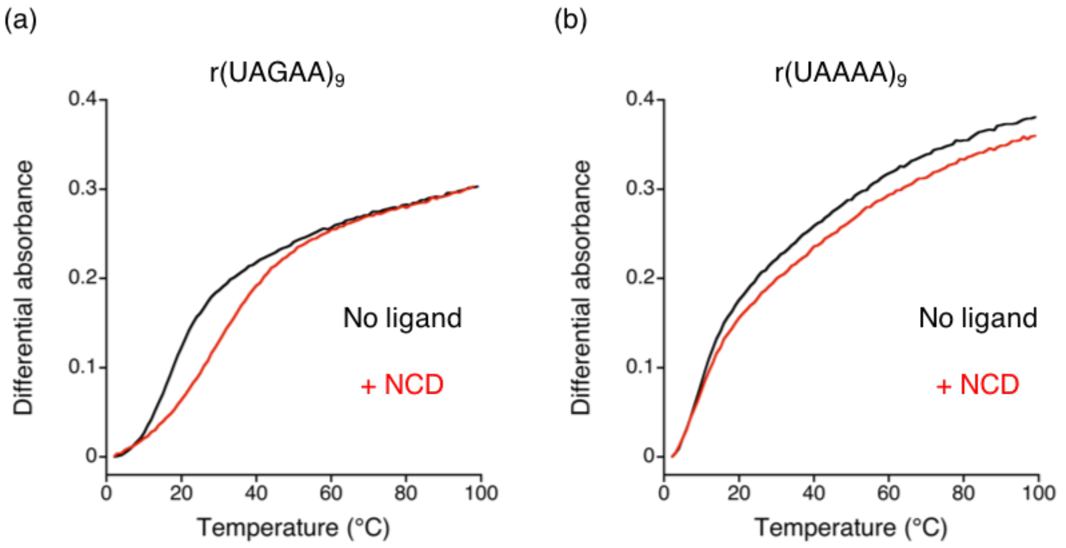

(c)

	T_m (°C)	
	No ligand	+ NCD
r(UGGAA)$_9$	N.D.	73.7 (1.1)
r(UAGAA)$_9$	N.D.	31.1 (1.0)
r(UAAAA)$_9$	N.D.	N.D.

Supplementary Figure 5

Thermal melting curves of (a) r(UAGAA)$_9$ and (b) r(UAAAA)$_9$ (2 μM) in the absence (black) and presence of NCD (red) in 10 mM sodium cacodylate (pH 7.0) containing 100 mM NaCl. Ligand concentration was 20 μM. (c) Table of T_m values of r(UGGAA)$_9$, r(UAGAA)$_9$, and r(UAAAA)$_9$ in the absence and presence of NCD (20 μM). The data are presented as the mean and SD is shown in parentheses (n = 3, independent experiments).

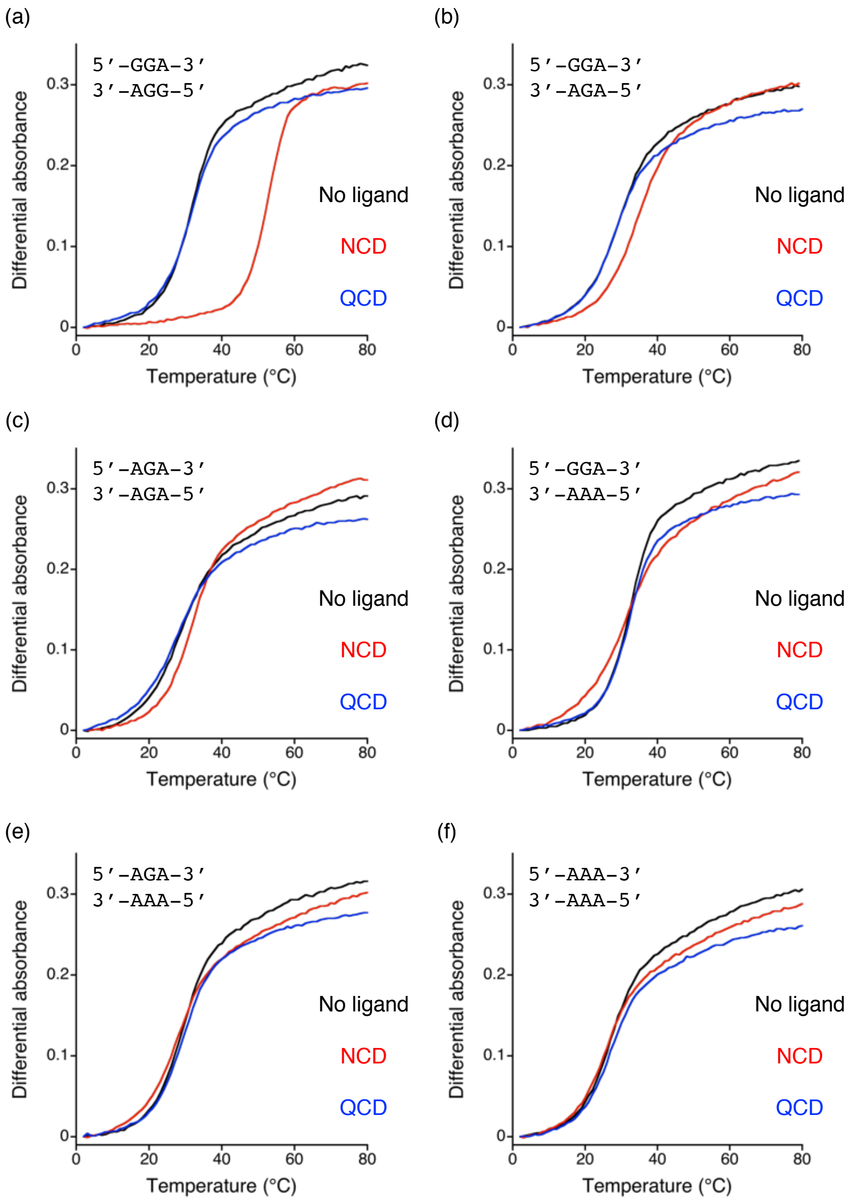

Supplementary Figure 6

Thermal melting curves of RRA/RRA internal loop-containing RNA duplexes (4 μM), r(GUACURRAACAUG)/r(CAUGURRAAGUAC) in the absence (black) and presence of NCD (red) or QCD (blue) in 10 mM sodium cacodylate (pH 7.0) containing 100 mM NaCl. (a) GGA/GGA, (b) GGA/AGA, (c) AGA/AGA, (d) GGA/AAA, (e) AGA/AAA, (f) AAA/AAA internal loop-containing RNA duplexes. Ligand concentration was 20 μM.

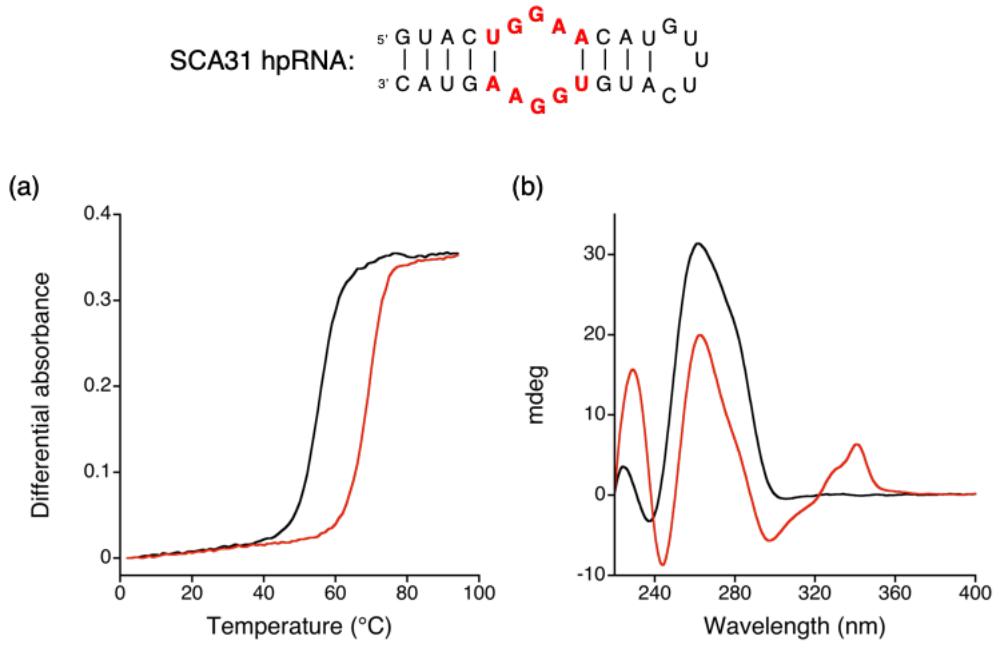

Supplementary Figure 7

(a) Thermal melting curves and (b) CD spectra of SCA31 hpRNA (4 μM), 5'-r(GUAC UGGAA CAUGUUUCAUG UGGAA GUAC)-3' without (black) and with (red) NCD (20 μM) in 10 mM sodium cacodylate (pH 7.0) containing 100 mM NaCl.

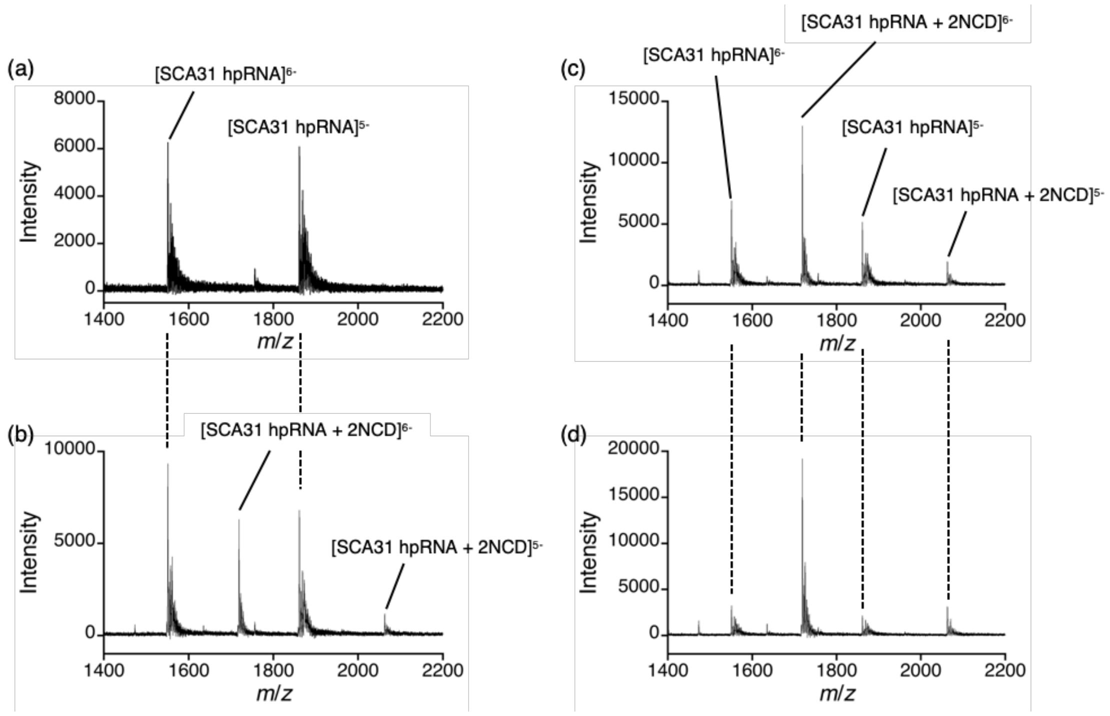

Supplementary Figure 8

ESI-TOF-MS analysis of SCA31 hpRNA (10 μM), 5'-r(GUAC UGGAA CAUGUUUCAUG UGGAA GUAC)-3' with NCD. Ligand concentration was (a) 0, (b) 5, (c) 10 and (d) 20 μM.

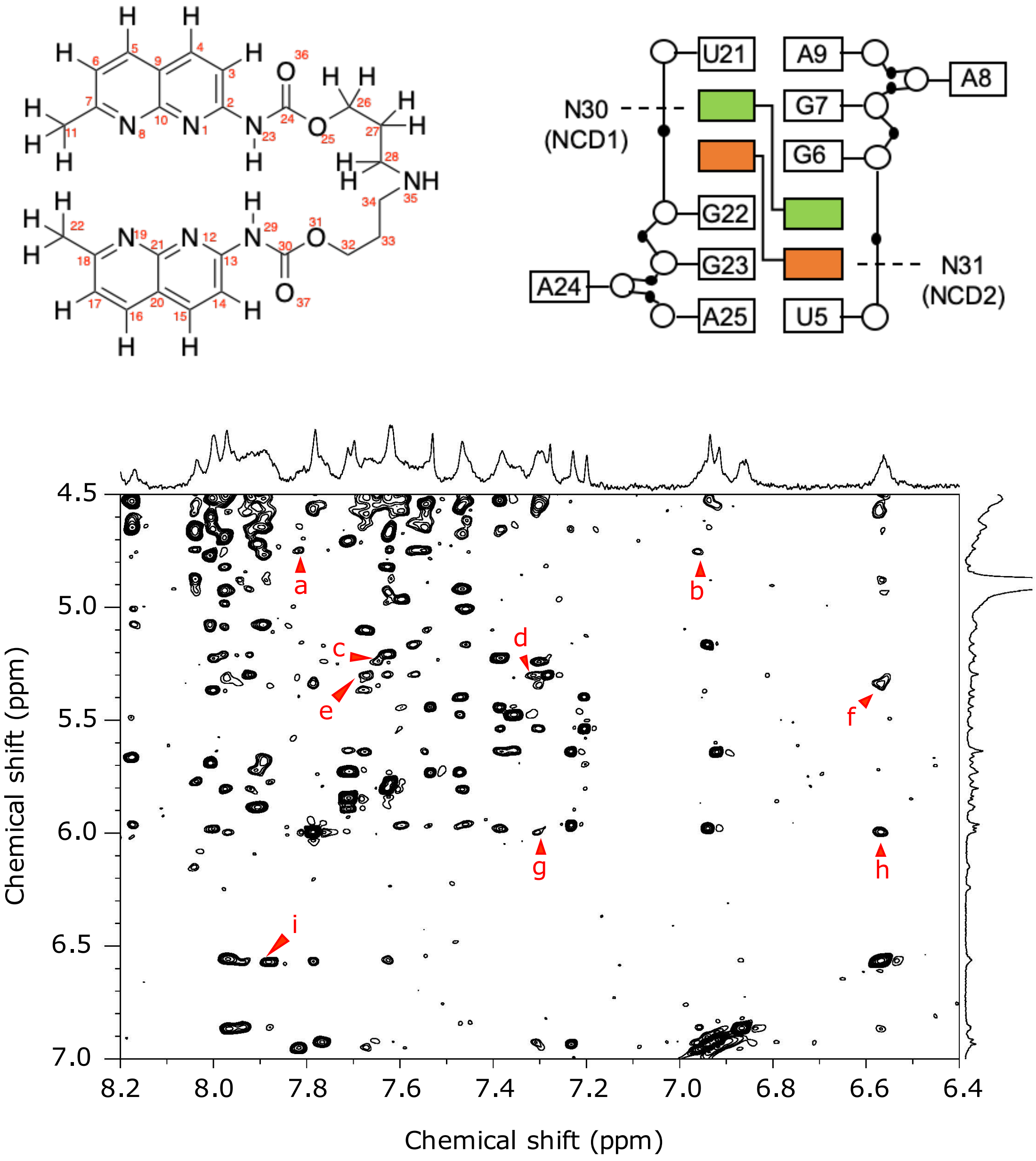

Supplementary Figure 9

Examples of intermolecular NOEs of SCA31 hpRNA and NCD (a) U5H5-N31H5, (b) U5H5-N31H6, (c) U21H1'-N30H4, (d) U5H1'-N31H3, (e) U5H1'-N31H4, (f) G22H2'-N31H14, G6H2'-N30H14, (g) G22H1'-N31H3, G6H1'-N30H3, (h) G22H1'-N31H14, G6H1'-N30H14, (i) N30H14-G6H8, N31H14-G22H8

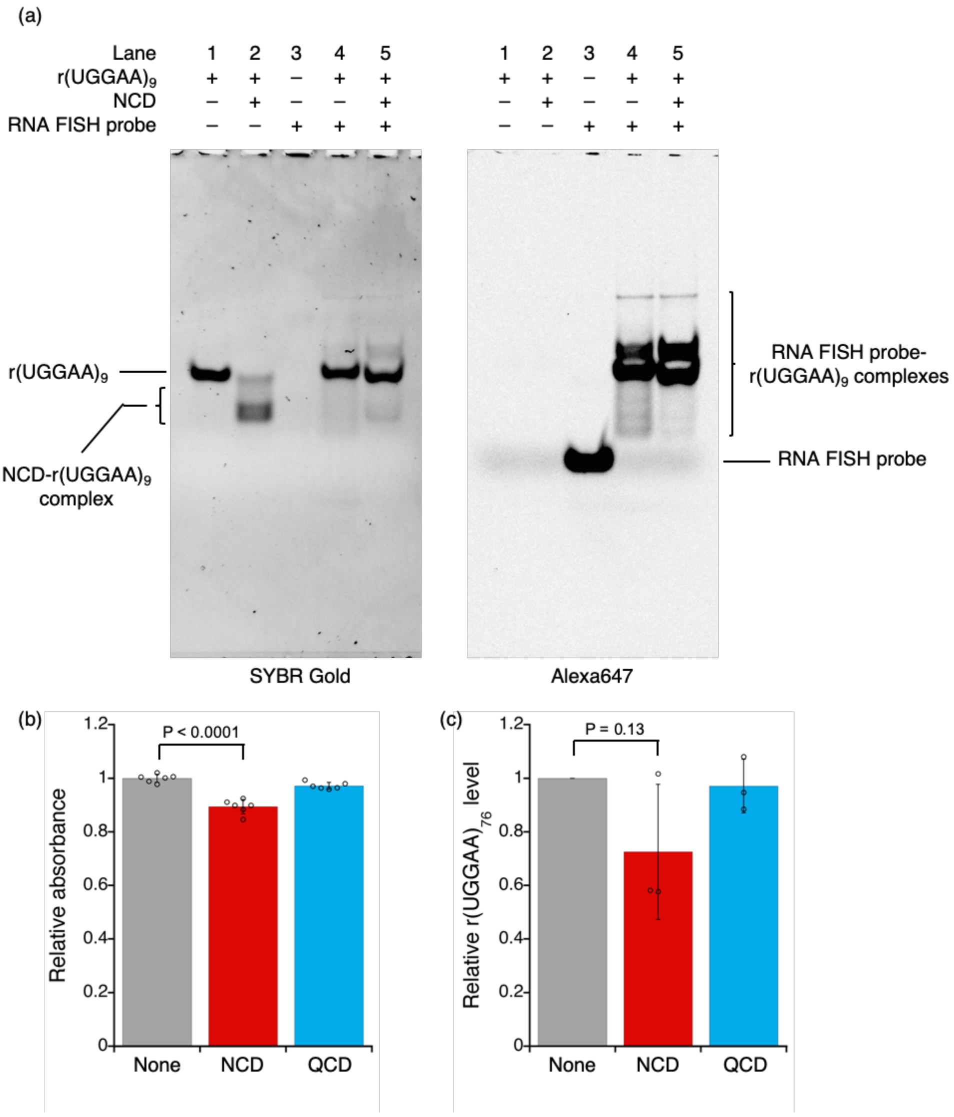

Supplementary Figure 10

(a) EMSA to confirm the interaction of r(UGGAA)$_9$ with RNA FISH probe in the absence and presence of NCD. r(UGGAA)$_9$ (200 nM) was incubated with RNA FISH probe (200 nM) in the absence and presence of NCD (2 μM) at 55 °C for 2 h. Bands were visualized by detecting SYBR Gold (left) and Alexa647 (right) with ImageQuant LAS 4000. RNA FISH probe: Alexa647-TTC5mCATT5mCCATTCCATT5mCCATTC5mCA, where underline is LNA and 5mC is 5-methylcytosine. (b) Cell viability assay and (c) Relative expression level of r(UGGAA)$_{76}$ in the absence and presence of ligands (5 μM). The *HPRT1* mRNA was used as an internal control. Data are shown as the mean ± SD (n = 6 for (b) technical replicates and 3 for (c) independent experiments); (Dunnett's multiple comparison test, two-sided).

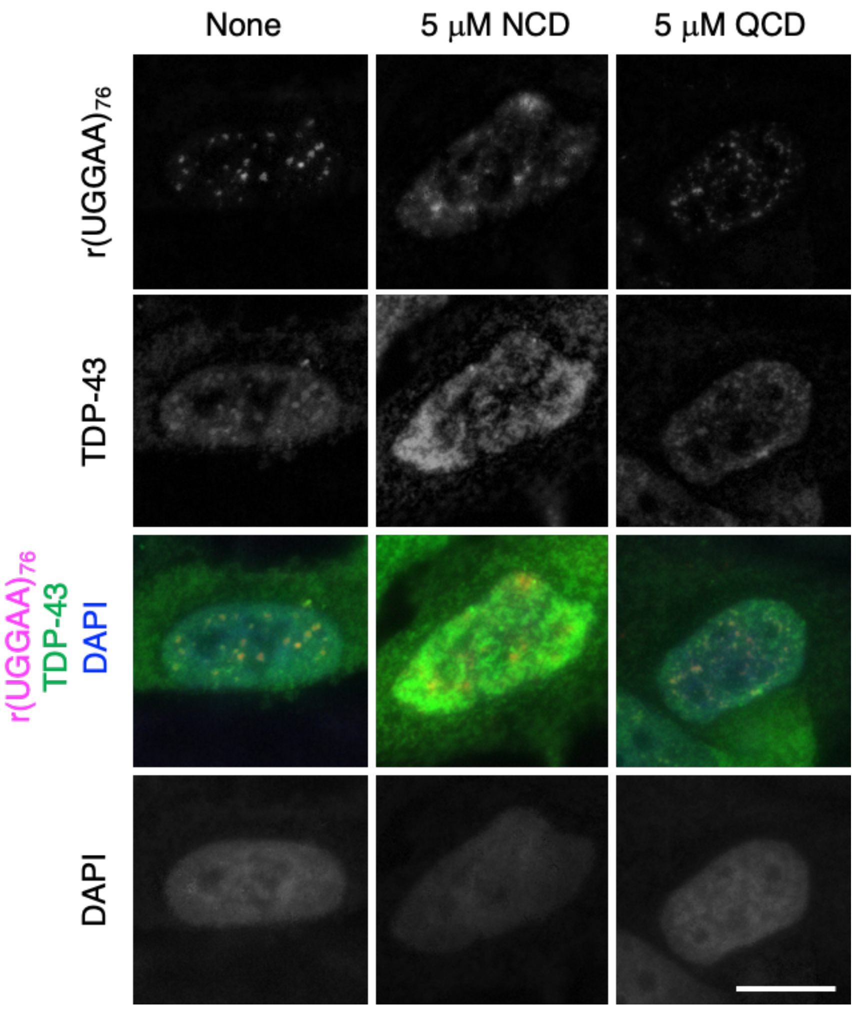

Supplementary Figure 11

RNA FISH and IF images of HeLa cells expressing r(UGGAA)$_{76}$ in the absence and the presence of NCD or QCD stained by r(UGGAA)$_{76}$-FISH and IF using anti-TDP-43 antibodies (n = 1 experiment). Scale bar: 10 μm. In the control and QCD-treated HeLa cells, r(UGGAA)$_{76}$ colocalized with TDP-43, while the colocalization of r(UGGAA)$_{76}$ with TDP-43 was not observed in NCD-treated HeLa cells. Concentration of Alexa647-labeled (TTCCA)$_5$ DNA/LNA probe was 10 nM.

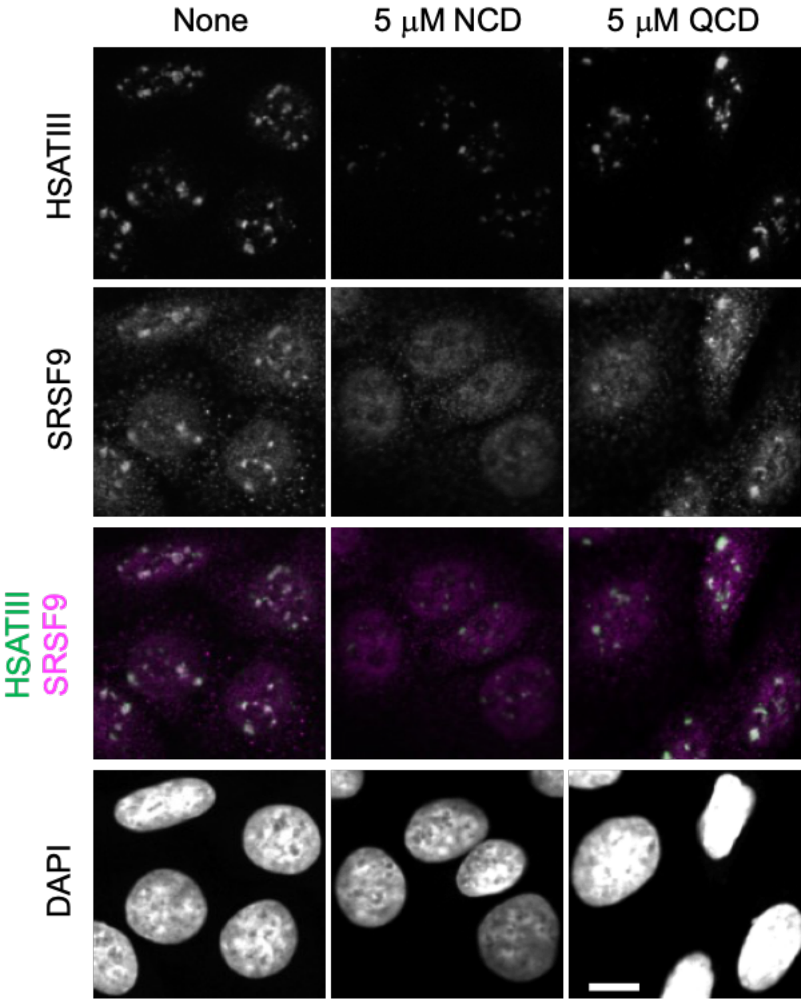

Supplementary Figure 12

RNA FISH and IF images of HeLa cells after thermal stress exposure in the absence and the presence of NCD or QCD stained by HSATIII-FISH and IF using anti-SRSF9 antibodies (n = 2, independent experiments). Scale bar: 10 μm. SRSF9 colocalized with nSBs in the control and QCD-treated HeLa cells. In contrast, the decrease of nSBs and the diffusion of SRSF9 throughout nucleoplasm were observed in NCD-treated HeLa cells.

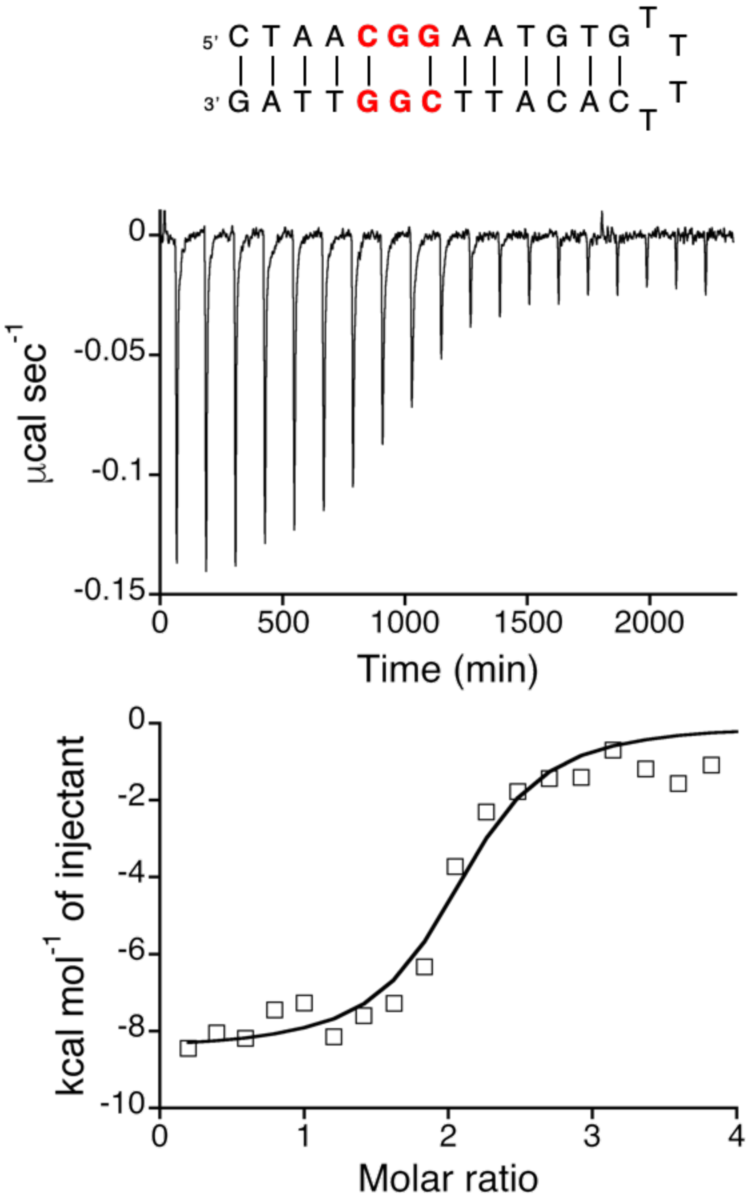

Supplementary Figure 13

ITC measurements for the binding of NCD to CGG/CGG-containing hairpin DNA.

Supplementary Table 1. NMR constraints and structure statistics.

Number of experimental restraints	
Distance restraints	192
Intra-residue	37
Sequential	53
Long range	21
Medium range	8
Hydrogen bonding distance	36
Ambiguous	37
Dihedral restraints	157
Planarity	14
Heavy-atoms r.m.s. deviation (Å)[a]	
All including NCD	1.966 ± 0.775
All including NCD (pairwise)	2.079 ± 0.359
Backbone of RNA	1.989 ± 0.787
Backbone of RNA (pairwise)	1.998 ± 0.396
Backbone of RNA without triloop, A8, A24	1.553 ± 0.616
Backbone of RNA without triloop, A8, A24 (pairwise)	1.696 ± 0.321
5-7,9,21-23,25 with NCD	1.069 ± 0.388
5-7,9,21-23,25 with NCD (pairwise)	1.013 ± 0.145

[a]Averaged r.m.s.d. between an average structure and the 10 converged structures were calculated. The converged structures did not contain experimental distance violation of >0.5 Å or dihedral violation >5°.

Supplementary Table 2. All-atom structure validation by MolProbity

1				
All-Atom Contacts	Clashscore, all atoms:		0	100th percentile* (N=1784, all resolutions)
	Clashscore is the number of serious steric overlaps (> 0.4 Å) per 1000 atoms.			
Nucleic Acid Geometry	Probably wrong sugar puckers:	2	6.90%	Goal: 0
	Bad backbone conformations#:	16	55.17%	Goal: <= 5%
	Bad bonds:	0 / 690	0.00%	Goal: 0%
	Bad angles:	1 / 1074	0.09%	Goal: <0.1%
Additional validations	Chiral volume outliers	0/144		
	Waters with clashes	0/0	0.00%	See UnDowser table for details
2				
All-Atom Contacts	Clashscore, all atoms:		0	100th percentile* (N=1784, all resolutions)
	Clashscore is the number of serious steric overlaps (> 0.4 Å) per 1000 atoms.			
Nucleic Acid Geometry	Probably wrong sugar puckers:	2	6.90%	Goal: 0
	Bad backbone conformations#:	16	55.17%	Goal: <= 5%
	Bad bonds:	0 / 690	0.00%	Goal: 0%
	Bad angles:	0 / 1074	0.00%	Goal: <0.1%
Additional validations	Chiral volume outliers	0/144		
	Waters with clashes	0/0	0.00%	See UnDowser table for detai
3				
All-Atom Contacts	Clashscore, all atoms:		0	100th percentile* (N=1784, all resolutions)
	Clashscore is the number of serious steric overlaps (> 0.4 Å) per 1000 atoms.			
Nucleic Acid Geometry	Probably wrong sugar puckers:	3	10.34%	Goal: 0
	Bad backbone conformations#:	15	51.72%	Goal: <= 5%
	Bad bonds:	0 / 690	0.00%	Goal: 0%
	Bad angles:	1 / 1074	0.09%	Goal: <0.1%
Additional validations	Chiral volume outliers	0/144		
	Waters with clashes	0/0	0.00%	See UnDowser table for details
4				
All-Atom Contacts	Clashscore, all atoms:		0	100th percentile* (N=1784, all resolutions)
	Clashscore is the number of serious steric overlaps (> 0.4 Å) per 1000 atoms.			
Nucleic Acid Geometry	Probably wrong sugar puckers:	1	3.45%	Goal: 0
	Bad backbone conformations#:	17	58.62%	Goal: <= 5%
	Bad bonds:	0 / 690	0.00%	Goal: 0%
	Bad angles:	0 / 1074	0.00%	Goal: <0.1%
Additional validations	Chiral volume outliers	0/144		
	Waters with clashes	0/0	0.00%	See UnDowser table for details
5				
All-Atom Contacts	Clashscore, all atoms:		0	100th percentile* (N=1784, all resolutions)
	Clashscore is the number of serious steric overlaps (> 0.4 Å) per 1000 atoms.			
Nucleic Acid Geometry	Probably wrong sugar puckers:	3	10.34%	Goal: 0
	Bad backbone conformations#:	14	48.28%	Goal: <= 5%
	Bad bonds:	0 / 690	0.00%	Goal: 0%
	Bad angles:	0 / 1074	0.00%	Goal: <0.1%
Additional validations	Chiral volume outliers	0/144		
	Waters with clashes	0/0	0.00%	See UnDowser table for details
6				
All-Atom Contacts	Clashscore, all atoms:		0	100th percentile* (N=1784, all resolutions)
	Clashscore is the number of serious steric overlaps (> 0.4 Å) per 1000 atoms.			
Nucleic Acid Geometry	Probably wrong sugar puckers:	1	3.45%	Goal: 0
	Bad backbone conformations#:	16	55.17%	Goal: <= 5%
	Bad bonds:	0 / 690	0.00%	Goal: 0%
	Bad angles:	0 / 1074	0.00%	Goal: <0.1%
Additional validations	Chiral volume outliers	0/144		
	Waters with clashes	0/0	0.00%	See UnDowser table for details
7				
All-Atom Contacts	Clashscore, all atoms:		0	100th percentile* (N=1784, all resolutions)
	Clashscore is the number of serious steric overlaps (> 0.4 Å) per 1000 atoms.			
Nucleic Acid Geometry	Probably wrong sugar puckers:	3	10.34%	Goal: 0
	Bad backbone conformations#:	14	48.28%	Goal: <= 5%
	Bad bonds:	0 / 690	0.00%	Goal: 0%
	Bad angles:	0 / 1074	0.00%	Goal: <0.1%
Additional validations	Chiral volume outliers	0/144		
	Waters with clashes	0/0	0.00%	See UnDowser table for details

8				
All-Atom Contacts	Clashscore, all atoms:		0	100th percentile* (N=1784, all resolutions)
	Clashscore is the number of serious steric overlaps (> 0.4 Å) per 1000 atoms.			
Nucleic Acid Geometry	Probably wrong sugar puckers:	3	10.34%	Goal: 0
	Bad backbone conformations#:	15	51.72%	Goal: <= 5%
	Bad bonds:	0 / 690	0.00%	Goal: 0%
	Bad angles:	0 / 1074	0.00%	Goal: <0.1%
Additional validations	Chiral volume outliers	0/144		
	Waters with clashes	0/0	0.00%	See UnDowser table for details
9				
All-Atom Contacts	Clashscore, all atoms:		0	100th percentile* (N=1784, all resolutions)
	Clashscore is the number of serious steric overlaps (> 0.4 Å) per 1000 atoms.			
Nucleic Acid Geometry	Probably wrong sugar puckers:	2	6.90%	Goal: 0
	Bad backbone conformations#:	18	62.07%	Goal: <= 5%
	Bad bonds:	0 / 690	0.00%	Goal: 0%
	Bad angles:	1 / 1074	0.09%	Goal: <0.1%
Additional validations	Chiral volume outliers	0/144		
	Waters with clashes	0/0	0.00%	See UnDowser table for details
10				
All-Atom Contacts	Clashscore, all atoms:		0	100th percentile* (N=1784, all resolutions)
	Clashscore is the number of serious steric overlaps (> 0.4 Å) per 1000 atoms.			
Nucleic Acid Geometry	Probably wrong sugar puckers:	1	3.45%	Goal: 0
	Bad backbone conformations#:	15	51.72%	Goal: <= 5%
	Bad bonds:	0 / 690	0.00%	Goal: 0%
	Bad angles:	0 / 1074	0.00%	Goal: <0.1%
Additional validations	Chiral volume outliers	0/144		
	Waters with clashes	0/0	0.00%	See UnDowser table for details
average				
All-Atom Contacts	Clashscore, all atoms:			100th percentile* (N=1784, all resolutions)
	Clashscore is the number of serious steric overlaps (> 0.4 Å) per 1000 atoms.			
Nucleic Acid Geometry	Probably wrong sugar puckers:	2.1		Goal: 0
	Bad backbone conformations#:	15.6		Goal: <= 5%
	Bad bonds:			Goal: 0%
	Bad angles:			Goal: <0.1%
Additional validations	Chiral volume outliers			
	Waters with clashes			See UnDowser table for details

The all-atom structure validation by MolProbity indicated 15.6 bad backbone conformations per structure in the UGGAA/UGGAA pentad and GUUUC loop regions. Probably wrong sugar puckers were 2.1 per structure which were distributed mainly in the same regions. The conformation of the GUUUC loop region was not well defined due to the intrinsic flexibility. For the UGGAA/UGGAA pentad, it is suggested that the interaction with NCD induced the conformational change in this region. It is noted that the clashscore was zero and no bad bonds and three bad angles were found for the ten structures.

Supplementary Table 3. Primer sequences used in RT-qPCR and semi-quantitative RT-PCR

HSATIII forward	5'-TATGAATTCAATCAACCCGAGTGCAATCGAA-3'
HSATIII reverse	5'-TATGGATCCTTCCATTCCATTGCTGTACTCG-3'
CLK1 exon 2 forward	5'-ATGAGACACTCAAAGAGAACTTACTG-3'
CLK1 intron 3 forward	5'-TGCCGCCCACTTGACGTTTCCAG-3'
CLK1 exon 6 reverse	5'-TTACTGCTACATGTCTACCTCC-3'
HSP-105 forward	5'-ATGTTTCTGCACAGAAAGATGG-3'
HSP-105 reverse	5'-TTTTGGGCTTTTTAGCTTCTGG-3'
TNRC6a forward	5'-GAATGTTACAAGACAAACGAAT-3'
TNRC6a reverse	5'-GTTGTGCTGCTGTGTTTCCA-3'
$r(UGGAA)_{76}$ forward	5'-CGAGCAGACATGATAAGATACATTGATG-3'
$r(UGGAA)_{76}$ reverse	5'-GCAATTGTTGTTGTTAACTTGTTTATTGC-3'
GAPDH forward	5'-ATGAGAAGTATGACAACAGCCTCAAGAT-3'
GAPDH reverse	5'-ATGAGTCCTTCCACGATACCAAAGTT-3'
HPRT1 primer	Housekeeping Gene Primer Set (TAKARA 3790)
